# In search of disambiguation: development of eye drop bottle sleeves to aid in identification and survey among possible users. A cross-sectional study

**DOI:** 10.1590/1516-3180.2020.0687.R1.27042021

**Published:** 2021-11-08

**Authors:** Ana Luiza Fontes de Azevedo Costa, Thiago Gonçalves dos Santos Martins, Vagner Rogério dos Santos, Paulo Schor

**Affiliations:** I MD, PhD. Pediatric Ophthalmology Fellow, UT Southwestern Dallas, Texas, United States.; II MD, PhD. Physician, Martins Clínica de Olhos, Rio de Janeiro (RJ), Brazil.; III PhD. Associate Professor, Department of Ophthalmology, Universidade Federal de São Paulo (UNIFESP), São Paulo (SP), Brazil.; IV MD, PhD. Head, Department of Ophthalmology, Universidade Federal de São Paulo (UNIFESP), São Paulo (SP), Brazil.

**Keywords:** Ophthalmic solutions, Medication errors, Social security, Usability, Textures, Sleeves, Aid for the blind

## Abstract

**BACKGROUND:**

Considerable numbers of individuals present low vision, blindness, illiteracy and other conditions that could possibly impair their identification of medications, such as eye drops. Through helping these individuals to identify their eye drops, they can achieve greater autonomy. Misidentification can be avoided through use of multisensory sleeves that can be adapted to most eye drop bottles. Correct use of eye drops is important for preventing progression of diseases like glaucoma that could potentially lead to blindness.

**OBJECTIVE:**

To develop bottle sleeves to aid in identification of eye drops and then interview a group of possible users to evaluate the acceptance of the solution.

**DESIGN AND SETTING:**

Cross-sectional survey performed at an ophthalmological clinic in São Paulo (SP), Brazil.

**METHODS:**

We describe the development of multisensory sleeves to assist in identification of eye drops. To assess the acceptance of this solution, we interviewed 18 patients who were currently using three or more types of eye drops.

**RESULTS:**

We developed four prototypes for eye drop bottle sleeves and conducted an acceptance test on them. Most of the patients who answered the survey about the sleeves were elderly. Most (95%) reported believing that the sleeves would help reduce the risk of mixing up eye drops with other medications that also dispense drops. They also believed that these would increase their autonomy in using eye drops.

**CONCLUSION:**

The solution presented was well accepted and may help increase safety in using eye drops through preventing misidentification.

## INTRODUCTION

Correct use of eye medications is very important for ensuring effective treatment of eye diseases. There is strong dependence on patient compliance and collaboration to ensure correct use. Incorrect use of these medications has already been demonstrated several times in the literature^1,2,3,4,5^ and can be characterized in terms of several flaws in the process of eye drop use, such as inadequate hygiene, incorrect identification of which eye drop bottles to use, incorrect instillation technique and lack of adherence to treatment. Among the difficulties that may impede the use of eye drops, incorrect identification of the eye drops to be used can be highlighted. This misidentification can occur in a hospital environment, or at a pharmacy at the time of purchase or during daily use.

Occurrences of mix-ups of ocular medications with each other or with substances not suitable for ocular use can be very harmful. In such situations, individual end up using a substance that, in addition to not treating their eye problems, can worsen their clinical condition through causing several side effects both locally and systemically.^6,7,8,9,10^ The similarities between bottles of eye medications and general substances can lead to an inadvertent misuse. Some substances are capable of causing severe eye burns since they do not have compatible pH.^11,12,13^ The opposite situation can also occur, when eye drops are used via a pathway other than ocular, thereby increasing their absorption and possibly generating serious effects, especially in individuals who are more susceptible due to comorbidities.^14^

Incorrect use of eye drops at home is very common, since most users, even those with experience in using eye drops, have the perception that they are using these drops correctly and do not recognize that they are making mistakes during this process. Some groups of people are at greater risk of using medications incorrectly: not only ocular but also systemic medications. Among these groups, the following can be highlighted: visually impaired individuals,^15^ users with low literacy,^16^ users of multiple types of eye drops,^6,7^ elderly people^17,18^ and disabled people with cognitive impairments such as dementia.^19,20^

The sense of vision is the most common way to interact with the environment and, therefore, the most common means for guiding decision-making regarding the chosen medication. Temporary or permanent visual loss increases the risk of misidentification, due to difficulty in reading the labels of eye drop bottles.^6,7,21^ According to the World Health Organization, there are 39 million blind people worldwide and 246 million people with low vision.^22^

Chronic eye conditions such as glaucoma may require the use of more than one type of eye drop for many years, or even for life. Glaucoma is the leading cause of irreversible blindness in the world, and its prevalence is increasing.^23^ The use of multiple types of eye drops may indicate greater severity of the condition under treatment, and this is also common in postoperative states. In both scenarios, correct use is critical. However, there is a greater risk of mix-up between medications due to the possible temporary visual changes and the increased number of medications to be used.

All over the world, the elderly population is increasing, and the prevalence of ophthalmic diseases is higher in this group.^24^ Advanced age is also usually accompanied by cognitive and visual impairment, which can affect the use of medications. One study showed that 66.3% of the individuals over 77 years old presented one or more limitations with regard to using their medications and, among these individuals, 31.8% were living alone and did not get help from others.^25^

Low literacy also limits the reading of labels and packaging.^16^ According to the Brazilian Institute for Geography and Statistics, the percentage of illiterate people in Brazil in 2017 was 7%, which represents around 11.5 million people.^26^ Worldwide, the United Nations Educational, Scientific and Cultural Organization (UNESCO) has estimated that in 2019, 100 million people between 15 and 24 years old were illiterate.^27^

Cognitive changes such as dementia create problems of adherence to treatment due to forgetfulness and altered perception of reality. The incidence of dementia increases significantly from the age of 65 years onwards.^28^

Some of these factors can overlap, and further increase the risk of misuse of eye medications. There are already some methods to help with identification of eye drops,^29,30^ but most of them have limitations of dependence on visual acuity, literacy or color vision. In the United States, the Food and Drugs Administration (FDA), supported by the American Academy of Ophthalmology, approved the use of different colors for eye drop bottle caps in 1996. This color difference helps many patients and healthcare professionals to better identify eye drops, but it is not so useful for those who have impairment of color vision, such as individuals with glaucoma or optic nerve diseases.^30^

Artificial intelligence also has the potential to improve the identification of medications, for both ocular and systemic conditions. Applications such as MobileNet, which uses deep learning, can be trained to automatically recognize labels.^31^ In addition, functions such as medication schedule reminders, with audible and visual alerts, can be added to show which medication should be used at a given time.

When there is a natural difference in the color of the ophthalmic solution, this becomes an additional factor to help differentiate between types of drops and prevent errors when there is no color vision deficiency. One study tested the impact of color differences to help in identifying eye drops and showed that there was a 64% improvement in correct identification of medications based only on analysis of the color of the substance of eye drops from identical bottles.^32^

In the present study, we detail the idealization and development of multisensory sleeves and evaluation of the acceptance of this new method to help reduce the risk of confusion between eye medications. Misuse of ocular medications is a subject that has only been poorly explored so far, but it is of great importance and needs to be addressed so that more solutions are proposed for increasing the safety of users. Specifically, we address eye drops that have extremely similar bottles and are, therefore, more prone to misidentification.

## OBJECTIVE

To develop eye drop bottle sleeves to aid in identification of eye drops and prevent mix-ups and misuse, and to interview a group of possible users to evaluate their acceptance of the solution.

## METHODS

### Study setting and design

We describe the idealization and development of four multisensory bottle sleeve prototypes containing different textures and odors, using low-cost flexible materials that are adaptable to most eye drop bottles commercially available in Brazil. This was a cross-sectional survey.

The prototypes chosen for the tests were sleeves made from orange-colored silicone material of thickness 2 millimeters, with different textures. To add the four different odors that were used, we left the sleeves immersed in essences for 24 hours before performing the test, to ensure that the odor would not dissipate before the test. The odors used were banana, chocolate, cinnamon and coffee, because these are well known by the Brazilian population.

After developing four sleeves with different textures and odors, we applied a verbal survey to a convenience sample of 18 adult individuals in an ophthalmological clinic in São Paulo (SP), Brazil. The investigator applied the survey to patients who had spontaneously sought emergency ophthalmological care for any eye problem and also to patients who were regularly scheduled for routine appointments at the ophthalmology service.

### Data source and patient characteristics

The survey was applied to individuals who were using three or more types of eye drops, regardless of the length of time for which they had been using these medications. All the individuals surveyed had firstly agreed to participate by signing a consent form. This study was approved by a local public university ethics committee (approval number 1326/2016; date: November 30, 2017).

The following exclusion criteria were applied: refusal to participate, presence of dementia that could be observed in the initial conversation, or use of less than three types of eye drops. The survey is shown in **Table 1**.

**Table 1.** Patient survey

1. What was the diagnosis that led to eye drop use?
2. Do you put in the drops yourself?
2a. If not you, who does this?
3. Have you ever used the wrong drop?
4. In your opinion, would the sleeves provide more autonomy?
5. In your opinion, would the sleeves reduce the risk of eye drop mix-up?
6. In your opinion, would the sleeves make it harder to squeeze the eye drop bottle to instill the drop?
7. In your opinion, which feature is more helpful? Texture, odors or both?

**Figure 1.**
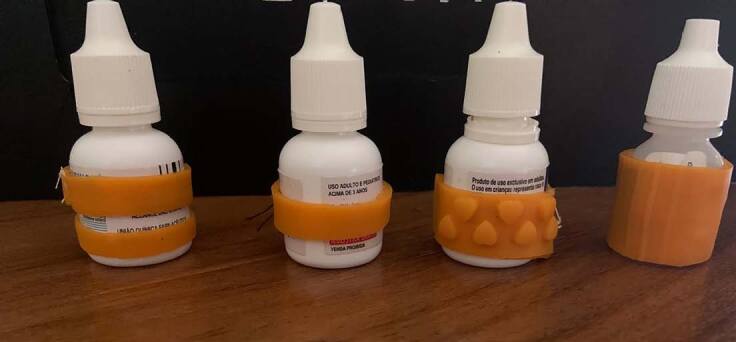
Prototype: the four types of sleeves produced and used in the survey.

The researcher gave the participants four bottles of eye drops produced by the same company, each with a different ophthalmological solution and wrapped in a sleeve with a different texture and odor (**Figure 1**). The participant was able to hold, test and analyze each eye drop bottle for as long as they wished, even during or after the questions in the survey.

## RESULTS

Four prototypes were produced from the orange silicone rubber material. This material had a thickness of 2 millimeters and was cut into strips of different widths. The ends of these strips were sewn together to form rings of different widths that would fit snugly around the eye drop bottles (always with a height outwards from the cylinder of the bottle of 2 millimeters). The bottle sleeves thus set up were as follows: one bottle with two flat rings, each 5 millimeters in width (odor of banana); one bottle a single smooth and flat strip of 5 millimeters in width (odor of cinnamon); one bottle with a strip of 1.5 centimeters in width with embossed hearts (odor of coffee); and one bottle with a strip of 1.5 centimeters in width with vertical grooves (odor of chocolate) (**Figure 1**).

Eighteen individuals without any profile of dementia who were using three or more types of eye drops analyzed these eye drop bottles with the sleeves and answered the survey verbally. The demographic data on the interviewees are shown in **Table 2**, together with the pathological conditions that motivated the use of the drops. Out of the total of 18 patients who participated in the survey, 44% of them were female. The average age was 70.1 years (range 54-96), and 61% of the respondents were elderly. The most common ocular pathological condition that had led to use of eye drops was glaucoma (62%).

**Table 2.** Demographic data on the questionnaire participants

Parameter	Number (%)
**Sex**	
Female	8 (44%)
Male	10 (56%)
**Level of education**	
Complete elementary school	2 (11%)
Incomplete elementary school	2 (11%)
Complete middle school	6 (33%)
Complete high school	4 (22%)
Higher education	4 (22%)
**Age**	
54-64 years	7 (39%)
≥ 65 years	11 (61%)
**Ocular pathological condition that required eye drops**	
Glaucoma	11 (62%)
Cataract, postoperative status	6 (33%)
Corneal ulcer	1 (5%)

The answers to the questions in the survey are shown in **Table 3**. Only three individuals (17%) declared that they needed someone else to help them use eye drops, and all of these individuals appointed family members to do this. The other 83% said they did not depend on others to use drops. When asked if there was any confusion in choosing and using the correct medication, eight (45%) denied having this problem, one (5%) reported not remembering and nine (50%) said they had already mixed up bottles previously.

**Table 3.** Answers obtained from the patients who were interviewed

Question	Yes	No
**Do you put in the drops yourself?**	15 (83%)	3 (17%)
Have you ever used the wrong drop?^*^	9 (50%)	8 (45%)
**Would the sleeves provide more autonomy?**	17 (95%)	1 (5%)
**Would the sleeves reduce the risk of eye drop mix-up?**	17 (95%)	1 (5%)
**Would the sleeves make it harder to squeeze the eye drop bottle to instill the drop?**	1 (5%)	17 (95%)
Which feature is more helpful?^**^		
- Texture alone	8 (45%)	
- Odor alone	2 (10%)	
- Both texture and odor together	7 (40%)	

* One patient (5%) could not remember whether the wrong drop had ever been used; **One patient (5%) said that neither feature was helpful.

When asked if, in their opinion, the sleeve would provide them with more autonomy, 17 (95%) answered yes, while one (5%) answered no. When asked if the sleeve would interfere at the time of instillation, one (5%) answered yes because the bottle would be stiffer, and 17 (95%) answered no. Only one individual (5%) replied that the sleeves would not help to better differentiate the eye drops and reduce the risk of confusion, while the other 17 (95%) believed that the sleeves would help in this regard. When expressing an opinion on the preference for attributes on the sleeve, one individual (5%) stated that they did not prefer any, eight (45%) preferred only the texture, two (10%) preferred only the odor and seven (40%) preferred both the texture and the odor (**Table 3**).

## DISCUSSION

The idealization and development of these prototypes for eye drop bottle sleeves was based on the observation that there was a need for assistance in identification of eye drops, especially among patients with visual problems, elderly people, illiterates and users of multiple types of eye drops.

The silicone material was chosen because it is inert, washable, safe and available at low cost. The choice of odors of banana, cinnamon, chocolate and coffee was intentional, as these odors form part of Brazilian culture and could thus further assist in the identification. The textures were chosen according to convenience, depending on the availability of the material used.

The acceptance test conducted was a type of usability test, which does not require a large sample to provide useful conclusions. The inclusion of individuals who were using three or more types of eye drops was essential, give that they are aware of the difficulties in dealing with multiple medications.

There have been reports in the literature regarding misidentification of ophthalmic medications in relation to substances that are not suitable for ocular use, even in the absence of use of multiple medications.^11–13^ In addition to having an impact on quality of life,^33^ use of multiple medications increases the risk of misidentification.^34^ Incorrect identification of medications is an underestimated problem that is not clearly apparent, due to the subtle effects of most misused eye drops.

Personal factors can also contribute to increased risk of misidentification. Among these factors, low levels of literacy,^16^ visual impairment,^15,21^ advanced age^35,36^ and cognitive changes^19,20^ can be highlighted.

Most of the individuals interviewed (61%) were elderly. Half of the individuals acknowledged that they had mixed up their eye drops previously. In such cases, this may have been due to their use of multiple types of eye drops and to the limitations of advanced age. This finding is probably an underestimate, given that misidentification can go unnoticed if it does not cause significant consequences.

Although the majority of the respondents considered that the bottle sleeves could increase their autonomy, the majority were also responsible for their use of eye drops themselves. The gain in autonomy would probably be more impactful for users of eye drops with low vision or who are blind, and those who are illiterate and more dependent on the help of others.

One of the concerns in adding sleeves to the bottles is the possibility that this could make it difficult to squeeze the bottle to instill the drop. Users of eye drops who present pathological conditions that affect manual movement may already have difficulty with instillation, and possibly would have greater difficulty with the addition of the sleeves.

The preference for the sense of touch had also been observed in a previous study,^37^ in which improved identification of eye drops when using sleeves was also demonstrated. The preference for touch can be explained by the ability to generate more effective memories than through the sense of smell. Smell-related memories are usually the result of memory about the feeling generated in response to the odor.^38^ In addition, olfactory changes are common among the elderly, with a prevalence of 60-75% in those over 80 years old,^39,40^ which may limit the utility of this characteristic for more advanced age groups.

Tactile acuity also suffers age-related decline, but it has been observed that this acuity is preserved in congenitally blind individuals and those undergoing intense tactile training.^41,42^

Healthcare professionals involved in patient care need to be able to identify which individuals are most at risk of using their medications incorrectly and should seek solutions to assist them. A study showed that women over 75 years old and elderly people with diabetes, hypertension and sequelae of stroke are at greater risk of developing limitations in activities of daily living. These patients need to be carefully evaluated and assisted in their use of medication.^43^

It was important to do the acceptance test among potential users of this solution, so that problems could be identified and changes made in order to improve the sleeves in accordance with the needs of some of the possible users.

The use of identification aids is a creative way to reduce the risk of errors in using eye drops. This may also help in improving adherence to treatment and the safety of the treatment, especially among patients who are most susceptible to error, such as the elderly and people with visual impairments, who represent a considerable portion of the population.

### Strengths and limitations of the study

Among the limitations of this study, we can mention the fact that a convenience sample was used and the fact that the component involving memory and cognition was not evaluated. More detailed studies with different populations of eye drop users could clarify which groups could gain greater benefit through use of these sleeves. Such studies could also promote greater adherence and enable assessment of possible clinical impacts.

Sleeves are a simple option that can be produced with flexible silicone material that is adaptable to most eye drop bottles. The idea of sleeves could be extrapolated to systemic medication bottles as well. In the United States, where prescription medication bottles are standardized, sleeves with a larger diameter could be useful for differentiating between bottles, especially in cases of polypharmacy, which is common in old age.

## CONCLUSION

Most of the patients using three or more types of eye drops who we interviewed (95%) believed that the sleeves could improve their autonomy in using the drops, and that the sleeves could reduce the risk of misidentification between bottles.

These multisensory sleeves for helping in eye drop identification were a well-accepted solution and may help increase safety in using eye drops.
